# Better together–*Salmonella* biofilm-associated antibiotic resistance

**DOI:** 10.1080/19490976.2023.2229937

**Published:** 2023-07-04

**Authors:** Adrianna Aleksandrowicz, Ewa Carolak, Agata Dutkiewicz, Aleksandra Błachut, Wiktoria Waszczuk, Krzysztof Grzymajlo

**Affiliations:** Faculty of Veterinary Medicine; Department of Biochemistry and Molecular Biology, Wrocław University of Environmental and Life Sciences, Wrocław, Poland

**Keywords:** *Salmonella*, biofilm, antimicrobial resistance, antibiotics, drug resistance mechanisms

## Abstract

*Salmonella* poses a serious threat to public health and socioeconomic development worldwide because of its foodborne pathogenicity and antimicrobial resistance. This biofilm-planktonic lifestyle enables *Salmonella* to interfere with the host and become resistant to drugs, conferring inherent tolerance to antibiotics. The complex biofilm structure makes bacteria tolerant to harsh conditions due to the diversity of physiological, biochemical, environmental, and molecular factors constituting resistance mechanisms. Here, we provide an overview of the mechanisms of *Salmonella* biofilm formation and antibiotic resistance, with an emphasis on less-studied molecular factors and in-depth analysis of the latest knowledge about upregulated drug-resistance-associated genes in bacterial aggregates. We classified and extensively discussed each group of these genes encoding transporters, outer membrane proteins, enzymes, multiple resistance, metabolism, and stress response-associated proteins. Finally, we highlighted the missing information and studies that need to be undertaken to understand biofilm features and contribute to eliminating antibiotic-resistant and health-threatening biofilms.

## Introduction

An estimated 1 in 10 people in the world fall ill every year from consuming contaminated food, with diarrhea being the most common form of these diseases. Salmonellosis is a major intestinal foodborne disease affecting approximately 200 million people globally, resulting in more than 200 000 fatal cases per year. Therefore, it is considered the cause of significant socio-economic losses and a real danger to human health, livelihood, and health care systems.^1^ According to the United States Department of Agriculture Economic Research Service (USDA ERS; The Economics of Food, Farming, Natural Resources, and Rural America), the economic impact of *Salmonella* exceeds 3 billion euros annually in the European Union.

The genus *Salmonella* consists of two species, *Salmonella bongori*, and *Salmonella enterica*, the latter of which is further divided into six subspecies. *S*. *enterica* subsp. *enterica*, comprising more than 1,500 different serovars, is mainly responsible for 99% of salmonellosis cases in humans and warm-blooded animals.^2^ Interestingly, only a few of these are clinically relevant. Each serovar is characterized by varying degrees of host adaptations and can be divided into one of the following groups: host-adapted specialists, for example, *Salmonella* Choleraesuis, which causes swine infections; host-restricted specialists, for example *S*. Typhi and *S*. Paratyphi, which are human pathogens; and host-unrestricted generalists such as *S*. Enteritidis and *S*. Typhimurium.^3^ The molecular basis of the infection process differs between generalists and specialists, and is determined by the bacterial genomic landscape. Generalists appear to rapidly induce disease symptoms and trigger the host immune system. Subsequently, they were eliminated from the body within a few weeks.^4^ In contrast, specialists may persist in the host body for decades.^5^ Most serovars are host-unrestricted pathogens and can infect different hosts based on the clinical patterns of salmonellosis. Two types of salmonellosis have been identified: typhoidal, caused by specialists *S*. Typhi and *S*. Paratyphi, and non-typhoidal (NTS), caused by other serotypes.^6^ The predominant NTS serovars in developing countries are *S*. Typhimurium and *S*. Enteritidis, which are increasingly resistant to many antimicrobial agents.^7^ Despite these variations, many *Salmonella* species share the capacity to induce latent, acute, or chronic illness, existing not only as planktonic cells but also as sessile, multicellular forms – biofilms attached to surfaces.

**Figure 1.**
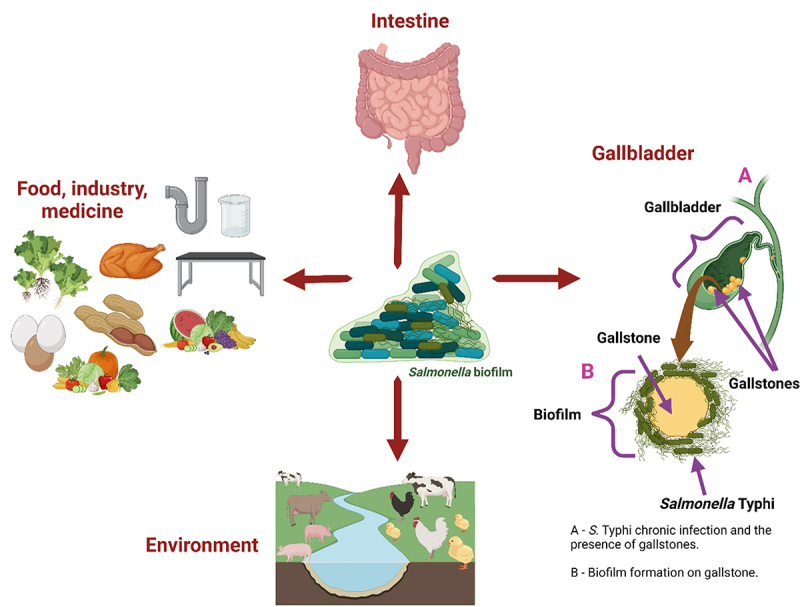
Presence of *Salmonella* biofilm in the environment and animals/humans.

Costerton introduced “biofilm” as a biological term in 1978.^8^ He described it as an interesting community formed in response to negative environmental stimuli such as temperature changes, oxygen, osmolarity, oxidative stress (bile), nutrient availability, or pH shifts. Biofilms may be formed on many biotic or abiotic surfaces, including the environment (lakes, rivers, soil, rock cover, aquatic plants, and sediments), food, water pipelines, medical devices, food processing equipment, animals, and humans^9^ (Figure 1).

**Figure 2.**
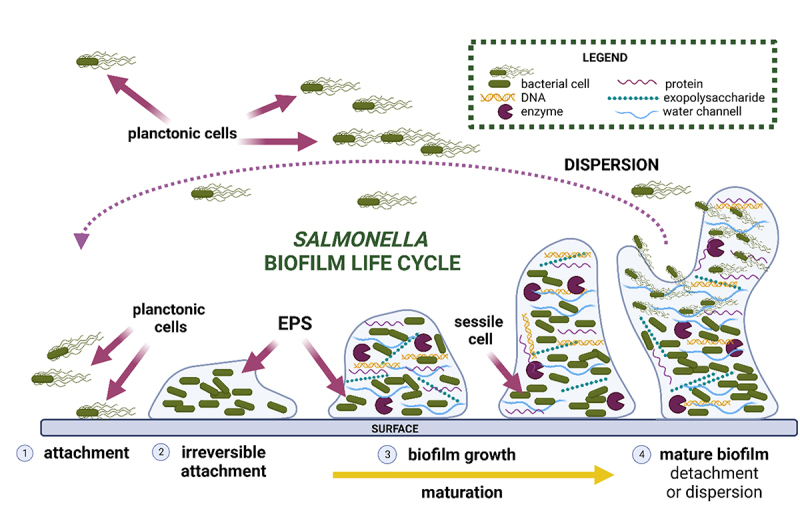
*Salmonella* biofilm life cycle.

Biofilm development can be divided into five stages: initial reversible attachment (1), irreversible attachment (2), maturation (3), and dispersion (4). The process begins with the initial contact between the planktonic bacteria and the surface, which is still reversible. The bacteria then form a monolayer and produce a protective extracellular matrix, consisting of extracellular polysaccharides, structural proteins, cell debris, and nucleic acids, collectively referred to as extracellular polymeric substances (EPS). The initial stages of matrix formation are dominated by extracellular DNA (eDNA), following by polysaccharides and structural proteins. The biofilm grows in a three-dimensional manner, and the attachment becomes irreversible. In the final stage, some cells in the mature biofilm detach and disperse into the environment as planktonic cells, potentially initiating a new cycle of biofilm formation^10^ (Figure 2).

The structure of these microbial communities ranges from homogenous, consisting of a single species of bacteria, to heterogeneous, comprising multiple species of microorganisms.^11^ Importantly, both gram-positive and gram-negative bacteria are capable of forming biofilms. Regarding *Enterobacteriaceae*, mechanism of biofilm formation is universal between species, and both *Salmonella* and *E. coli* utilize similar pathways to form this structure: biosynthesis of type 1 fimbriae, flagella, lipopolysaccharide, as well as production of curli fimbriae, transmembrane transport and a variety of transcriptional regulators.^12^ Although there are many similarities between both bacteria, a few differences were also identified. For example, the divergent utilization of *tomB* which deletion aids biofilm of *Salmonella*, but impairs its formation in *E. coli*.^12^

One of the most essential properties of bacteria that are an integral part of biofilms is increased resistance to environmental stress, including the presence of xenobiotics.^13^ In comparison to the planktonic state, biofilms are even 1000 times more resistant to antibiotics.^14^ In a biofilm, horizontal gene transfer (HGT) often takes place, which results in bacteria acquiring resistance to several groups of antibiotics.^15^ Consequently, changes in gene expression profiles are believed to closely resemble those of planktonic cells in the stationary growth phase.^16^ Moreover, if there is a sub-inhibitory concentration of antibiotics in the external environment surrounding the biofilm, the bacteria may also become resistant to the substances in this case.^17^ All this can lead to the development of multidrug resistant (MDR) strains that exhibit increased resistance to nearly all classes of antibiotics.^9,15,18^ Additionally, the extracellular polymeric substances (EPS) produced by bacteria limit the penetration of antibiotics into the structure of the biofilm.^11^ This phenomenon is caused mainly due to the cellulose and curli fimbriae present in it, which play a role not only in adhesion to the surfaces but also in imparting resistance to the biofilm by enabling tight packing of cells covered with a hydrophobic net.^18^

The race between *Salmonella* multidrug resistance and antibiotic (and other antibacterial substances) use and development is still ongoing. Infections and risk of loss in industries such as medicine, water, food, and energy, where these bacteria can cause significant damage, are still significant worldwide problems.^19^ Therefore, further studies are needed for a more effective *Salmonella* biofilm control.

## Mechanisms responsible for the antibiotic resistance of *Salmonella* in biofilms

**Figure 3.**
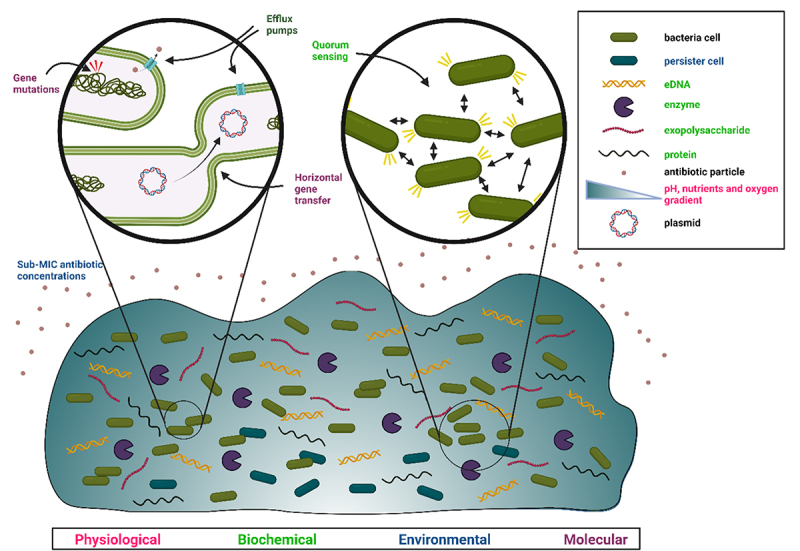
Graphical representation of main resistance mechanisms in *Salmonella* biofilm. Each color represents different factors, and mechanisms regulated by it: physiological (pink), biochemical (green), environmental (blue), and molecular (purple).

The use of antimicrobials to fight against biofilms can reduce the number of bacterial cells in the structure but does not lead to complete eradication, contributing to the development of chronic and recurrent infections. The main resistance mechanisms of *Salmonella* biofilms are regulated by (1) physiological, (2) biochemical, (3) environmental, and (4) molecular factors^20^ (Figure 3).

### Physiological factors

Pathogenic bacteria are exposed to unfavorable conditions in the host organism. This stress often triggers the bacterial adaptive response, which may alter gene expression, induce mutations, or promote a resistant lifestyle such as biofilm formation.^21^ A biofilm is a physical barrier protecting bacteria from antimicrobial agents, as the first stage of spreading of the particles is diffusion in a dense environment.^22^ Therefore, the biofilm matrix is not homogeneous and has a gradient of oxygen, pH, and nutrients (Figure 3). A gradient of oxygen is formed, with the highest concentration in the outer layers of the biofilm, steadily decreasing deeper into the biofilm matrix. A lack of oxygen may be one of the causes of persister cell formation. As a consequence of uneven oxygen distribution, a pH gradient occurs inside the biofilm matrix. The lower pH present in the inner layers of the biofilm is a result of lack of oxygen, which induces anaerobic respiration, as well as difficulties with the removal of metabolic products, such as CO_2_. Differences in pH may also interfere with the penetration of some antibiotics depending on their type. Lower pH was also found to promote the expression of genes associated with resistance mechanisms, such as multidrug resistance pumps, in some *Enterobacteriaceae*.^21^ It is still unclear whether the fluctuation of pH is a direct cause of some of the resistance mechanisms or the aftermath of other mechanisms.^23^

Along with oxygen transportation inside the biofilm matrix, it is also difficult for nutrients to penetrate deeper layers. Therefore, a microgradient of metabolic substrates forms. This slows down the growth of bacteria embedded in biofilms, making them more resistant to antibiotics as they often target the metabolic cycle of cells.^24^

### Biochemical factors

Similar to that of biofilms of other species, *Salmonella’s* biofilm contains several types of EPS, such as polysaccharides, proteins, nucleic acids, and matrix.^25^ All these components play a significant role in biofilm formation and antimicrobial resistance. Although the exact contribution of each EPS type is yet to be determined, several attempts have been made to investigate it. It was determined that curli fimbriae, cellulose, O-antigen capsule, and colanic acid are involved in biofilm formation, as the deletion of these factors impairs biofilm formation.^26^ Curli fimbriae had the greatest effect on biofilm formation. This is probably because the adhesive properties of these fibers, along with cellulose, have been shown to play crucial roles in cell aggregation, adhesion to surfaces, and biofilm formation.^25,27,28^ Another study showed that the O antigen capsule, colanic acid, and Vi-antigen affect biofilm-mediated responses to oxidative stress.^27^ Moreover, both colonic acid and Vi-antigen present in the matrix were able to protect deletion mutants and wild-type bacteria from H_2_O_2_ in cooperation with catalases. Interestingly, not all polysaccharides displayed community functions in co-cultures with different mutants. O-antigen-deficient mutants were not compensated by co-culture with wild-type bacteria, and co-culture of wild-type and curli fimbriae mutants, both resistant to H_2_O_2_, resulted in the sensitization of both strains.^29^ O-antigen capsules were found to play a role in protecting bacteria from desiccation stress, as it was shown that deletion of the *agfD* gene responsible for formulation of capsules resulted in significant loss of viability in freeze-dried samples of *S*. Typhimurium (31% for wild type and 1.3% for *agfD* mutant).^30^

Quorum sensing is a method of communication between cells according to the density of the bacterial population using so-called autoinducers.^31^ It is driven by the production of particular molecules and sending them out of cells by either passive or active transport. Bacteria then recognize the particles via dedicated receptors and change their gene expression. Among the autoinducers produced by *Salmonella*, few have been linked to biofilm formation and antimicrobial resistance induction.^32,33^

Quorum sensing is also one of the factors that induces HGT in some bacteria. N-acyl homoserine lactone was found to play a role in biofilm formation by *S*. Typhimurium.^32^ Quorum sensing (*luxS*) has also been linked to the activation of gene expression on *Salmonella* pathogenicity island-1, which is responsible for motility, virulence, and biofilm formation.^33^

*Salmonella*, a gram-negative bacteria, is less susceptible to antibiotic treatment than gram-positive bacteria owing to its double membrane and efflux systems.^34^ Although the outer barrier slows down the antibiotic particles, the pumps can transport the antimicrobial agents out of the cell and send quorum sensing signals.^35^ In *S*. *enterica*, several efflux pumps are encoded in its genome as well as in plasmids carried by the bacteria.^36^ In *S*. Typhimurium, at least ten pumps have been experimentally identified.^36,37^ The aforementioned pumps are divided into five superfamilies of transporters: 1) the ATP-binding cassette superfamily (ABC transporters), 2) the major facilitator superfamily (MFS), 3) the drug/metabolite transporter (DMT), 4) the multidrug and toxic compound extraction (MATE), and 5) the resistance nodulation-cell division (RND) superfamily.^38^

### Environmental factors

One of the ways in which bacteria resist antibiotics is by spontaneously forming persister cells mainly in the deeper layers of the biofilms. These cells are dormant and their metabolism is muted. Therefore, antimicrobial agents that interfere with metabolic reactions have little effect on the persister cells.^39^ Persister cells are often associated with chronic diseases and can turn back into regular cells and their planktonic form to further infect host organisms. Biofilms are a way for bacteria to withstand higher concentrations of antimicrobial agents, and the presence of antibiotics may also induce the formation of biofilms. Some antibiotics, such as nalidixic acid, spectinomycin, tetracycline, and neomycin, were found to induce biofilm formation in *S*. Infantis cultures at sub-minimum inhibitory concentrations (MIC).^40^ A study on *S*. Typhi showed a similar effect to that of cefetoxime.^41^ Although some antibiotics may promote biofilm formation, others have the opposite effect, as shown in a study with *S*. Typhimurium.^42^

### Molecular factors

Gene expression in biofilms is substantially different from that in the planktonic form. Apart from changes in gene regulation, mutations can aid in the resistance of bacteria to antimicrobial agents. Gene mutations that occur in bacteria may be part of an adaptive response to stressful environments.^2^ For example, a mutation in *soxRS*, a gene directly involved in the control of the AcrAB-TolC multidrug efflux system and indirectly with limitation of antimicrobial uptake, has been found in several serovars of *Salmonella*, as well as in other gram-negative bacteria.^43,44^ HGT plays a major role in antibiotic resistance. Microbes can share additional genes between themselves and, therefore, help each other fight antimicrobial agents. Plasmids are shared from one cell to another by a conjugation pilus and can carry multiple antibiotic-resistance genes simultaneously.^45,46^ It has been shown that antibiotic-resistance genes can be transferred between different species, from *Escherichia coli* to *Salmonella*.^47^ Although there are many studies regarding the differences in gene expression and gene mutations in *Salmonella*, their effects on biofilm resistance and formation have not been thoroughly studied. Therefore, we attempted to gather all available data on the topic in the following chapters.

Taken together, these factors provide protection against antibiotics, osmotic shifts, metallic cations, oxidative stress, UV, desiccation, biocides, and even from washing or cleaning. In addition, biofilms enable competition for nutrients and space.^48^ Biofilms provide a wide range of benefits for *Salmonella* and are a threat to humans. These bacterial consortia play a negative role in chronic human infections. *Salmonella* spp. adheres to and forms biofilms in the gastrointestinal tract.^9^ Furthermore, *S*. Typhi can form robust biofilms on the cholesterol gallstones present in the gallbladder.^49^ Consequently, the human immune system is mobilized to fight pathogens that are significantly straitened for biofilms as compared to planktonic cells. Considering this, biofilms are an exceptional adaptation mechanism that allows bacteria to survive in unfavorable conditions; therefore, it is a crucial threat to public health.

## Upregulation of antibiotic resistance-associated gene expression in *Salmonella* biofilms

Molecular biology techniques have revolutionized the study of bacterial pathogenicity processes, including the formation of biofilms. These techniques allow researchers to investigate the genetic and molecular mechanisms underlying bacterial biofilm formation, providing insights into the factors that contribute to bacterial virulence.

Besides quantitative PCR (qPCR), microarray analysis and RNA sequencing are the most common strategies, which allow the simultaneous monitoring of thousands of genes, providing a comprehensive quantitative view of the gene expression pattern that occurs during biofilm formation.^50,51^ More recently, CRISPR-Cas technology has emerged as a robust tool for investigating bacterial pathogenicity, allowing for targeted editing of bacterial genomes and enabling researchers to investigate the function of specific genes involved in virulence.^52^

Although these techniques are useful for identifying mutations and genetic changes in bacterial populations, they do not provide information on the functional consequences of these changes or how they affect bacterial fitness. Therefore, recent studies have focused on another powerful strategy to characterize the genetic mechanisms of resistance: experimental evolution coupled with whole-genome sequencing (WGS).^53,54^ By propagating bacterial populations in the presence of antibiotics, this strategy allows for the selection of clones capable of surviving antibiotic exposure. WGS of these populations or clones reveals the genetic causes of the resistance phenotype and sheds light on the interaction between selection, chance, and historical contingency in microbial populations. Previous studies used this approach to track the evolution of bacterial resistance to antibiotics in different contexts. This method has been used to study the evolutionary dynamics of combining antibiotics, deploying them sequentially, and adapting to changes in temporal or spatial drug concentration.^55,56^ The study revealed that resistance evolution is influenced by divergent genetic changes resulting from different genetic backgrounds and drug exposure.^54^ It is worth noting, that the evolution of decreased susceptibility to antibiotics in biofilm and planktonic populations is not identical, as different mechanisms and trajectories are involved. Mutations in genes that code for antibiotic targets are commonly found in planktonic populations evolved in the presence of antibiotics, while mutations in efflux and metabolism genes are often observed in evolved biofilm populations.^57^ Growth in well-mixed planktonic cultures tends to select for high-level resistance under subinhibitory concentrations of antibiotics, while growth in spatially structured biofilms favors mutants with lower levels of resistance.^58^ However, this is not always the case when using stepwise increasing or lethal concentrations of antibiotics during evolution. Evolved biofilm populations maintain a higher diversity than corresponding planktonic populations, which may protect against a negative selection of less fit-resistant mutants.^59^ Mutations in different genes may lead to similar phenotypes, suggesting that the fundamental mechanisms behind reduced biofilm susceptibility could be similar for different classes of antibiotics and organisms.

**Figure 4.**
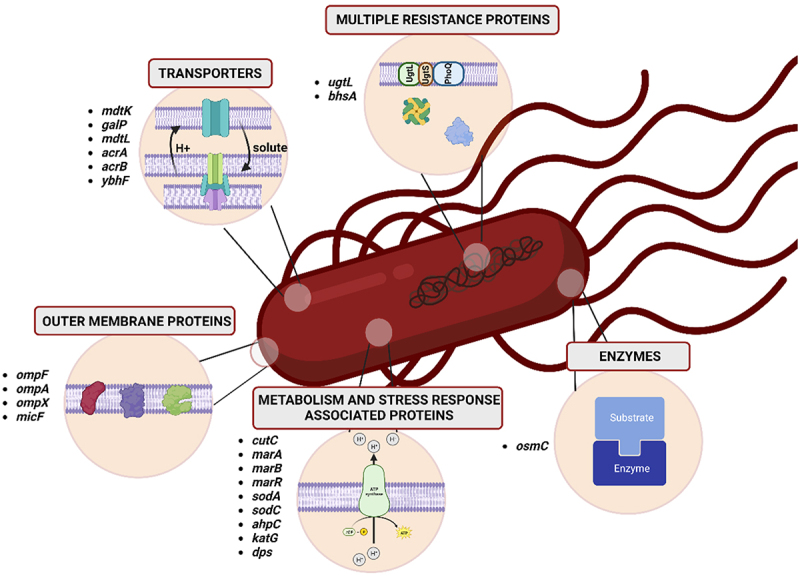
Groups of differentially upregulated genes in *Salmonella* biofilm/aggregate cells.

Experimental evolution can help elucidate the interplay of resistance, tolerance, and persistence behind the reduced antimicrobial susceptibility of biofilms. However, identifying complex patterns of mutations, gene expression, and metabolism will require an interdisciplinary and holistic approach. In this study, we utilized recently published data to investigate the drug-associated gene expression profile of biofilm-forming *S*. *enterica*. We focused on identifying the genes that are upregulated in multicellular aggregates compared to planktonic cells (Table 1; Figure 4).

**Table 1.** Selected differentially upregulated genes and their functions in *Salmonella* biofilms/aggregate cells.

Gene	General function category	Expanded function Category	Function description	Methodology	Ref.
*mdtK* *mdtL*	**MEMBRANE TRANSPORT**	Major Facilitator Superfamily/Multidrug Resistance	Functions as a Na(+)/drug antiporter; inactivation in *Vibrio cholerae* results in susceptibility to fluoroquinolones	RNA-seq	^51^
*galP*		Major Facilitator Superfamily/Multidrug Resistance	Galactose/proton symporter	RNA-seq	^60^
*tolC*		Resistance-nodulation-division family transporters	Outer membrane porin - efflux pumps a.o. antibiotics, such as quinolones, aminoglycosides, chloramphenicol, and tetracycline; part of AcrAB-TolC efflux pump	Flow cytometry	^61^
*acrA* *acrB*		Resistance-nodulation-division family transporters	Part of AcrAB-TolC efflux pump	qRT-PCR	^62^
*ybhF*		ABC type transporter	Resistance to tetracyclines and cephalosporin	RNA-seq	^51^
*ugtL*	**MULTIPLE** **RESISTANCE**	Antimicrobial resistance	Resistance to a variety of antimicrobial peptides; SlyA regulon; PhoPQ regulon	RNA-seq	^51^
*bhsA*		Influencing biofilm through hydrophobicity and stress response	Multiple stress resistance protein BhsA	RNA-seq	^63^
*ompF* *ompA* *ompX*	**OUTER** **MEMBRANE** **PROTEINS**	Outer Membrane Protein	Outer Membrane Proteins - OmpF, OmpA, OmpA	qRT-PCR; microarray hybridization	^64–66^
*micF*		Stress response	Control of the *ompF* expression	qRT-PCR	^66^
*cutC*	**ENERGY** **METABOLISM, XENOBIOTIC DEGRADATION AND STRESS** **RESPONSE**	Copper metabolism/Xenobiotic metabolism process	Copper homeostasis	RNA-seq	^51^
*marA* *marB* *marR*		Fumarate hydratase class II	Multiple antibiotic resistance protein MarA; multiple antibiotic resistance protein MarR; Multiple antibiotic resistance protein MarB, repressor for MarA	RNA-seq; qRT-PCR; hybridization to the arrays	^63,66,67^
*sodA* *sodC*		Oxidative phosphorylation	Superoxide radicals degradation; Xenobiotic metabolic process	RNA-seq	^51^
*ahpC*		Stress response	Xenobiotic metabolic process; AhpCF protects aerobic, phosphate-starved cells from oxidative damage; the AhpC component carries out the actual reduction of the hydroperoxide substrate	RNA-seq	^60^
*dps*		DNA protection during starvation	Protection from multiple stresses: oxidative stress, the stress of exposure to visible light, under conditions of fatty acid starvation	microarray hybridization	^65^
*katG*	**ENZYMES**	Stress response	Xenobiotic metabolic process	RNA-seq	^60^
*osmC*		Organic hydroperoxide reductase OsmC/OhrA	Osmotically inducible; putative resistance protein [*S*. typhimurium LT2]	microarray hybridization	^65^

### Transporters

#### Efflux pumps

The first group comprises genes related to membrane transport encoding MFS, multidrug resistance, and RND efflux pumps. Efflux pumps are a key element of resistance for gram-negative bacteria, especially planktonic cells, which are more exposed to antibiotics than bacterial consortia, forming a biofilm structure. Therefore, it is not surprising that there is a tendency to reduce the expression of genes responsible for the efflux of substances from cells in the biofilm structure. Biofilm, which is a mechanical barrier limiting the access of harmful substances from the external environment, reduces the need for cells to develop a system of efflux pumps. However, several genes associated with efflux pumps encoding structural or regulatory proteins are upregulated in biofilm cells.^51,60–66^

#### MFS (major facilitator superfamily)

MFS is a family of secondary transport proteins and one of five efflux pumps described in prokaryotes. They are primarily engaged in the uptake of sugars; however, numerous MFS proteins are also implicated in drug efflux systems, and they increase antibiotic resistance in gram-negative bacteria, including *Salmonella*. Many microbial genomes contain MFS transporters that typically function as single-component pumps capable of moving small solutes through the inner membrane.^68^ Five of the nine known MDR efflux pumps (AcrAB, MdtK, AcrEF, EmrB, and MdfA) in *S*. Typhimurium genome can export quinolones when overexpressed.^69,70^ In clinical *S*. Typhimurium, an active MDR efflux pump has recently been identified as the factor for the main fluoroquinolone-resistant mechanism. It was also shown that deletion of efflux pumps such as *acrD, acrEF, emrAB, macAB, mdfA, mdsABC, mdtBC*, and *mdtK* reduced the ability of *S. enterica* serovars to produce biofilms.^71^

Based on comparative analysis, two efflux pump-coding genes *mdtK (ydpH) mdtL* and *(yidY)*, were significantly upregulated in the biofilm cells compared to that in the planktonic cells of *S*. Typhimurium isolated from stainless steel surfaces.^51^ MdtK is present in all *Enterobacteriaceae* families and shares 90% similarity with its homologs in *Salmonella* and *E. coli*, indicating its high conservation among the *Enterobacteriaceae* member family.^70^ By inhibiting the accumulation of antimicrobial dipeptides through quorum sensing, the MdtK pump may be crucial for the efflux of dipeptides in *Salmonella* cells. It was demonstrated that MdtK imparted acriflavine, doxorubicin, and norfloxacin resistance.^72^ Antibiotics and several antimicrobial peptide components with antibacterial action are extruded by the superfamily MdtK from the PhoP/PhoQ system.^73^ This observation has also been reported for efflux dipeptides, such as alanylglycine, in *E. coli*.^74^ Despite antibiotic resistance, *Salmonella*‘s capacity to produce biofilms is reduced in *S*. Typhimurium when MdtK is deleted or its activity is chemically inhibited.^71^ Another gene involved in multidrug resistance is *mdtL*. Studying global gene expression in *E. coli*, *mdtL* showed a high 18-fold upregulation in biofilms compared to that in the planktonic form.^75^ The multidrug resistance efflux protein MdtL belongs to the drug/H+ antiporter (DHA) subfamily of the MFS superfamily. Its overexpression led to 2-fold higher chloramphenicol resistance.^76^ In *Salmonella* (except for serovar *S*. Typhimurium), *mdtL* is directly adjacent to *Salmonella* genomic island 1 (SGI1).^77^ Considering that *E. coli* and *Salmonella* are closely related, a similar observation can be expected in *Salmonella* biofilms.

Different gene expression patterns between planktonic cells and multicellular aggregates *S*. Typhimurium 14,028 biofilm was also found in the case of another representative of the MFS superfamily, the galactose permease GalP.^60^ It has been shown that overproduction of inner membrane proteins, like carbohydrate permeases, can alter antibiotic resistance mediated by those pumps in *E. coli* and *Salmonella* cultures, using a non-PTS carbohydrate as the only carbon source.^78^ One such permease, GalP, induces exclusion-dependent galactose permease that transports sugar through the bacterial membrane, and also transports glucose, whose expression is triggered by galactose.^79,80^ When a non-PTS carbohydrate was the only carbon source, *S*. Typhimurium showed increased susceptibility to paraquat, indicating that the overproduction of permeases such as GalP caused by the presence of non-PTS sugars interfered with the efflux pumps linked to paraquat resistance.^78^

#### RND (resistance-nodulation-cell division)

AcrAB-TolC is the best-characterized RND system in *Enterobacteriaceae* including *E. coli*, *Salmonella*, and *Klebsiella pneumoniae*. The RND efflux system family has the greatest clinical significance as it is associated with multidrug resistance.^61,81^ Clinically important is the induction of *acrAB-tolC* by bile, fatty acids, and cationic peptides.^81^ This pump is composed of a TolC channel, with AcrB and AcrA proteins positioned in the inner membrane and periplasmic space.^82^ It removes a wide range of substances from the bacterial cell, including antibiotics such as quinolones, aminoglycosides, chloramphenicol, and tetracycline, which provide resistance to a broad spectrum of antibiotics.^61,83^ This system is present both in planktonic bacteria and biofilms, but changes in the expression of its components are visible. Such differences are related to the *tolC* gene, which encodes the TolC channel, with higher *tolC* expression observed in the planktonic form of *S*. Typhimurium. Significantly, changes in gene expression were observed not only between planktonic and biofilm bacteria but also between single-species biofilms and those composed of a multispecies bacterial community. Upregulation of *tolC* was observed in mixed biofilms composed of two *S*. Typhimurium strains and one *E. coli* strain.^61^ Moreover, in the presence of eugenol, the expression *of acrA* and *acrB* in biofilm cells was higher than that in planktonic cells.^62^

*S*. Typhi biofilm is also characterized by a higher expression of genes responsible for energy production and conversion: *marA*, *marB*, and *marR*. MarA is a global regulator that affects *acrAB* expression as well as many other genes that play a role in bacterial response to stress.^67,84^ MarA acts mainly as an activator and directly or indirectly regulates many genes, including *acrAB* and *tolC*.^67,85^ The MarR protein, on the other hand, is a repressor of the *marAB* operon.^86^ MarR blocks MarA transcripts when there are no signals from the environment induced by phenolic compounds, antibiotics, or oxidative stress. Derepression of the *marAB* operon results in the expression of *marA*, which encodes the global transcriptional activator MarA, which in turn is subject to positive feedback regulation and represses MarR, allowing *marA* to be active.^81^ Perera and Grove demonstrated that the Mar regulon is associated with antibiotic resistance and stress response.^87^ Increased MarA expression was observed in the biofilm form of S*almonella* in the presence of triclosan biocide. Triclosan increased the expression of *marA* and *acrAB*, which was activated by *marA*. In stationary cells, no significant effects on the expression of *marA* and *acrA* were observed.^66^ According to Prieto et al., who demonstrated that the genes *marA* and *marB* were upregulated when exposed to bile, the Mar regulon also controls bacterial virulence factors, such as cell wall proteins and surface adhesins.^88^ Therefore, it is conceivable that the Mar regulon prepares the *Salmonella* cells for possible antibiotic resistance or for stressors such as bile utilized to form biofilms. Another reason may be that some proteins have multiple roles. For example, the efflux pump in pathogenic organisms can pump antibiotics out of the cytoplasm of cells and into the surrounding environment, in addition to serving as a mechanism to expel toxic chemicals from inside the cells to the external environment. Therefore, the Mar regulon may perform two tasks: pumping out bile or antibiotics.

#### ABC (ATP-binding cassette)

One of the largest superfamily of transport system are ABC transporters which use energy from ATP hydrolysis to transport substrates across the membrane. Representative of this group is the YbhFSR transporter consisting of three proteins: YbhF, YbhR, and YbhS. YbhF is an ATP-binding component, whereas the next two are membrane components. In this case, a 2-fold upregulation of *ybhF* gene was observed in *S*. Typhimurium biofilms compared to those in the planktonic cells.^65^ It has been shown that the main antibiotic substrates for YbhFSR are tetracyclines such as tetracycline, oxytetracycline, chlortetracycline, and doxycycline.^89^ Additionally, deletion of *ybhF* resulted in increased sensitivity to cefoperazone (a third-generation cephalosporin) and a decreased growth rate compared to that of the wild type.

### Outer membrane proteins

Drug transport through the outer membrane is another bacterial mechanism that plays a role in resistance to environmental stresses like antibiotics.^88^ Porins are associated with antibiotic resistance by participating in the passive transport of antibiotics through the outer membrane of gram-negative bacteria.^90^ Therefore, when threatened with such substances, microbial cells reduce the expression of genes that lead to the formation of porins or hinder the penetration of harmful compounds into cells. Changes in the expression of porins – reduction in porin levels and point mutations within them – favor the emergence of resistance.^91^ The better-studied porins in the *Enterobacteriaceae* family are the highly conserved OmpF and OmpC proteins^92^ whose expression is regulated by OmpR in a manner that depends on the osmolarity of the environment.^93^ By considering the correlation between the expression of genes and their regulators, we suggest that overexpression of *ompF* and *ompC* could be associated with the elevated expression of *ompR*. It is one of the regulatory genes for the biofilm and an important virulence factor for *Salmonella*. Its deletion prevents biofilm formation by repressing pili and cellulose.^94^ *ompR* is also involved in cell adhesion as one of the genes that regulate curli biosynthesis as OmpR controls the transcription of *csgD* whose product regulates the expression of curli subunits CsgA and CsgB.^95^

Another gene involved in the regulation of OmpF is *micF*, a stress response gene whose protein controls the expression of the *ompF* gene found in *E. coli*, *Salmonella*, and related bacteria, to ensure resistance to carbapenems or β-lactams.^96,97^ OmpF is a nonspecific porin that is involved in the transport of antibiotics.^90^ Through OmpF porins, antibiotics, such as β-lactams and fluoroquinolones, enter the cell; therefore, *ompF* mutations in gram-negative bacteria can cause resistance to these antibiotics.^96^ The expression of the *micF* gene in

*S*. Typhimurium in the presence of triclosan was higher than that in the 16sRNA controls, but was similar for both biofilm and planktonic forms.^66^ However, in a study conducted by Chin et al. in which NGS was used to determine the transcriptome of *S*. Typhi in planktonic and biofilm forms, a higher expression of *micF* in the biofilm form not exposed to any chemical compound was noted.^63^ OmpC is an important factor in preserving membrane integrity but also participates in the transport of antibiotics.^90^ Loss of OmpC causes *Salmonella* resistance to cephalosporins.^98^ However, its expression in biofilm is reduced compared to planktonic form.^63^

Overexpression of another OMP was observed in *E. coli* biofilms. The OmpA protein was 4-fold overexpressed in the biofilms compared to its expression in the planktonic form in proteome studies, as confirmed by immunoassay enzyme-linked immunosorbent assay (ELISA) and western blotting. OmpA is a conserved OMP of *Enterobacteriaceae* with several different functions.^64^ OmpA allows the passive transport of many small chemicals, including antibiotics, because it is a nonspecific porin. Deletion of OmpA has been shown to increase antibiotic susceptibility in several cases. *E. coli* deletion mutants showed increased susceptibility to antibiotics, such as β-lactams, glycopeptides, amphenicols, and lincosamides, whereas in *S*. Typhimurium, OmpA has been shown to increase bacterial survival when exposed to two β-lactam antibiotics, ceftazidime and meropenem.^99^ Additionally, in the case of *Acinetobacter baumannii*, increased susceptibility to β-lactams was noted.^100^ The protective role of OmpA against the harmful effects of β-lactam antibiotics is probably due to the maintenance of outer membrane stability.^99^

Another OMP gene *ompX* showed an almost 5-fold upregulation in *S*. Typhimurium.^65^ OmpX is a small porin, and similar to OmpF, it is involved in the permeability control of β-lactams and fluoroquinolone antibiotics. During exposure to drugs and environmental stress, the expression *of ompX*, encoding the outer membrane protein, is increased.^101^ This protein was originally described in *Enterobacter cloacae* but is also present in various enterobacterial species, including *E. coli*, *Enterobacter aerogenes*, and *S*. Typhimurium.^101–103^

### Metabolism and stress response-associated proteins

In addition to genes related to biofilm development and xenobiotic transport, genes involved in stress tolerance, oxidative stress, heat shock, cell envelope stress, putative stress, osmotic stress, acid tolerance responses, and DNA replication and repair were also upregulated in biofilms.^104^

It was established that *S*. Typhimurium could utilize two possible routes to gain nitrate from the host: (1) direct nitrate absorption from the environment via nitrate transmembrane transporters NarK and NarU, which are encoded by the *narK* and *narU* genes, respectively, and (2) synthesis of nitrate from NO and O2 by a flavohemoprotein.^105,106^ NarU was shown to function as a nitrate/nitrite antiporter, or, more likely, a nitrate/H+ or nitrite/H+ channel.^107^ Although there is no evidence of the contribution of this gene to the antimicrobial resistance of *Salmonella*, it was demonstrated that chlorhexidine resulted in significantly higher transcript levels of *narU* (5-fold).^108^ During nutritional deprivation or extremely slow growth, NarU, which is abundant in the stationary phase, confers a selection advantage. Following chlorhexidine therapy, the increased transcription of a gene related to nitrate absorption may indicate the activation of processes resembling anaerobic respiration.

The second group consisted of genes associated with reactive oxygen stress (ROS)-inducing response: *ahpC* and *katG* which showed increased expression in multicellular aggregates relative to planktonic cells isolated from *S*. Typhimurium 14,028 cultures.^60^ ROS is a weapon against lipids, proteins, and nucleic acids, causing several types of intracellular damage.^109^ Bacterial oxidative stress responses involve the action of enzymes, small compounds, and regulatory proteins that directly detoxify or defend against ROS. AhpC scavenges endogenous H_2_0_2_ at the physiological level in *E. coli* whereas catalase KatG provides protection at higher H_2_0_2_ concentrations.^110^ The substrates of *S*. Typhimurium AhpC are small hydroperoxides and organic hydroperoxides, including alkyl hydroperoxides, which can be intracellularly produced from unsaturated fatty acids and nucleic acids.^111,112^ Mature biofilm bacteria are essentially starved for iron, as evidenced by very low catalase levels and increased Mn-containing superoxide dismutase activities.^113^ Thus, the low expression of catalase in biofilms mediated by alterations in overall iron metabolism, relative to planktonic bacteria, may be one factor that allows for increased expression of AhpCF in response to H_2_O_2_. The level of OxyR-regulated AhpC remains high in mature biofilms, and deletion of the *ahpC* gene appears to promote early biofilm formation by inducing exopolysaccharide production. Furthermore, the addition of exogenous H_2_O_2_ or antioxidants modulates biofilm formation by altering exopolysaccharide production.^114^ KatG is referred to as hydroperoxidase I or catalase peroxidase. KatG has been shown to decrease susceptibility to aminoglycosides. According to these findings, kanamycin helped the peroxidase reaction by acting as an electron donor, bringing KatG’s oxidized ferryl intermediates back to their resting state.^115^

Increased expression levels of genes associated with metabolism and xenobiotic biodegradation, *cutC* and *sodAC*, in *S*. Typhimurium, isolated from stainless steel surfaces, were also demonstrated.^51^ CutC, a cytosolic protein of 248 amino acids, is responsible for copper homeostasis.^116^ In 2008, the United States Environmental Protection Agency (US EPA) recognized copper as the first antimicrobial metal. In *in vitro* assays, solid copper surfaces killed 99.9% of microorganisms within two hours of contact.^117^ A mutant lacking *cutC* was more susceptible to copper than its parental strain, supporting the theory that *cutC* is a cytoplasmic copper-binding protein.^118^ Interestingly, this protein contains a putative copper-binding motif and can confer copper tolerance to both *cutC* and *cutF* mutants of *E. coli*.^118^ Cadmium exposure causes oxidative stress, and the enzymes that are able to detoxify superoxide anions contribute to cadmium resistance. The capacity of bacteria to resist cadmium toxicity is attributed to the activity of the two cytoplasmic superoxide dismutases, the manganese-containing MnSOD, encoded by *sodA*, and the iron-containing FeSOD, encoded by *sodB*.^119^ The importance of periplasmic Cu- and Zn-SOD in *Salmonella* pathogenesis is underscored by the existence of two distinct and unlinked *sodC* genes. The first of the discovered genes is designated *sodCI* and appears to be encoded on a cryptic A-like bacteriophage. The second locus, *sodCII*, was more closely related to the *E. coli sodC* gene. Because the combination of mutations in both *sodC* genes provides a higher reduction in virulence in mice than single mutations in either *sodC* gene alone, both SodC proteins appear to be functionally significant. The biological function of *sodA* in antimicrobial resistance was also investigated. SodA deactivation resulted in a reduced bacterial growth rate, low SOD activity, and high sensitivity to reactive oxygen species and chicken serum.^104^

Dps (DNA-binding protein from starved cells) is a stress-related protein similar to ferritin, and it showed an almost 6-fold increase in expression in *S*. Typhimurium biofilms.^65^ Because of its DNA-binding properties, it protects DNA from oxidative damage and is also able to protect cells during periods of nutrient deficiency and other environmental stresses, including ultraviolet and gamma irradiation, metal toxicity (iron and copper), thermal stress, and acid/base shocks.^120,121^ Dps is common in starved cells, and mutants lacking Dps exhibit significant changes in protein synthesis in response to starvation stress. It forms very stable complexes with DNA and the complex formation does not require sequence specificity.^121,122^ When examining the response of the *dps* deletion mutant of *S*. Enteritidis, hypersensitivity to four different antibiotics was observed compared to the wild strain: two quinolones (nalidixic acid and norfloxacin), an aminoglycoside (streptomycin), and rifampicin, which is a semisynthetic drug. However, these differences were completely reduced when the cultures were supplemented with an iron chelator.^120^

### Multiple resistance proteins

Upregulation of genes associated with microbial resistance, including *ugtL* and *bhsA*, was observed in biofilms compared to their expression in the planktonic form.^51^ UgtL is a PhoQ accessory protein that contributes to monophosphorylated lipid A formation.^123^ It was demonstrated that the *Salmonella ugtL* mutant strain has a deficiency in gut colonization, using a mouse model of streptomycin-induced diarrhea. Outer membrane integrity and sensitivity to magainin 2, an alpha-helical CAMP, were also diminished in the *ugtL* mutant strain. Additionally, it was discovered that PhoP-activated *ugtL* is necessary for polymyxin B resistance.^124^

BhsA is a protein encoded by a porin gene that regulates membrane permeability in response to ROS generated by H_2_0_2_ and HOCl.^125^ It is also referred to as the STY1254 gene, which is one of the most highly upregulated genes in *S*. Typhi biofilms.^63^ Zhang et al. indicated that the *bhsA* gene in *E. coli* increases the stickiness of the protein to the membrane, enabling it to adhere to surfaces during biofilm formation. The scientists demonstrated that the deletion of the *bhsA* gene in *E. coli* led to increased biofilm formation because bacteria were unable to adhere to the surface, leading them to produce more biofilm matrix as a form of defense.^126^ Additionally, Salazar et al. discovered that *bhsA* contributes to *S*. Typhimurium adhesion to the surface of glass, polystyrene, spinach leaves, and tomato fruit.^127^ The two genes, *bhsA* and STY1255, may be responsible for the adherence of *S*. Typhi to the surface of polypropylene tubes, enabling the development and maintenance of biofilms, according to their activities and the findings of transcriptome investigation.^63^ In addition, the absence of *bhsA* increases the resistance of *E. coli* to carbon monoxide-releasing molecule-2 (CORM-2), which acts as an inhibitor of bacterial growth, even in MDR strains.^128^

### Enzymes

Another group of genes upregulated in *Salmonella* biofilms encodes enzymes. The gene *osmC* of *E. coli* belongs to a family of homologs distributed across a wide range of species, including *Salmonella*. Several studies on the regulation of these genes have demonstrated that they are induced by a variety of stressors. OsmC is also activated in *Salmonella* biofilms.^65^ The involvement of OsmC in defense against oxidative stress was proposed in a study investigating the survival of *E. coli* cells in media with different NaCl concentrations, in which the hydroperoxide activity of OsmC was subsequently demonstrated.^129^ However, the biochemical functions of the OsmC-like proteins remain unknown. Its homolog in *Xanthomonas campestris*, *ohr*, is involved in protection against organic hydroperoxides.^130^ This was also the case for *osmC* in *E. coli*. Indeed, the *osmC* mutant exhibited a higher sensitivity to H_2_O_2_.^129^

### *Biofilm-forming* Salmonella*: a threat to health and environment and strategies for prevention and control*

The gene expression profile of bacteria in biofilms differ significantly from that of their planktonic counterparts. This is due to the complex, three-dimensional structure of the biofilm and the presence of an extracellular matrix that can alter genetic patterns. One of the most significant implications of altered gene expression in biofilms is their increased antimicrobial resistance. These problem appears in healthcare settings, where biofilms form on medical devices and lead to increased risk of treatment failure.^131^ Furthermore, altered genetic patterns have important consequences in the spread of infectious diseases. Biofilms can provide a protective environment for bacteria, allowing them to evade the immune system and persist in the host even in harsh conditions.^132^ This persistence results in chronic infections that can be not only difficult to treat but also promote the formation of new, more virulent strains of bacteria. Moreover, biofilms facilitate the horizontal transfer of genetic material, including virulence factors and antibiotic-associated genes, which can lead to the spread of these traits to other microorganisms.^133^

While *Salmonella* is a type of enteric pathogen, its biofilm is a significant concern in the food processing and packaging industries as it can contaminate both fresh and processed food products.^134^ This corruption occurs either directly during processing or indirectly when uncompromised products come into contact with previously contaminated machinery or surfaces. It was shown that *Salmonella* adhere to different materials used in industrial settings, including stainless steel, glass, and polystyrene, and persist in such environment for a long time.^135^

In addition to causing foodborne illness, *Salmonella* biofilms contaminate water systems.^136^ Pathogens can survive in water for extended periods, allowing corruption of drinking water and recreational water sources such as lakes and swimming pools. What is more, *Salmonella* alters the physical and chemical properties of the surface, resulting in changes in nutrient cycling and water quality.^137^

Although sanitation practices are implemented to disinfect possible sources of cross-contamination, *Salmonella* biofilms are more challenging to disinfect compared to their planktonic counterparts, making such practices less effective. It leads to the need for more aggressive cleaning agents, such as biocides which have a detrimental environmental impact and deleterious consequences for human health.^138^ Additionally, the use of biocides could act as selection pressures for increased microbial resistance to antimicrobial compounds, and the sanitizers may lose effectiveness gradually. This phenomenon, known as antimicrobial cross-resistance, is considered as one of the most challenging to public health worldwide.^139^

Addressing this problem requires a multifaceted approach that considers both socioeconomic and bacterial perspectives. There are two main strategies to overcome biofilms: inhibiting their formation or dispersing established biofilms.^140^ Preventing biofilm development involves inhibiting bacterial adherence to surfaces and their subsequent growth. Surface modification, such as imprinting 3D patterns or targeting physicochemical properties, can be effective at preventing early attachment.^141^ Another approach is pre-conditioning surfaces with surfactants that inhibit bacterial adherence.^142^ Improving sanitation and hygiene practices in industries is therefore crucial to prevent biofilm-associated *Salmonella* contamination. Awareness campaigns, education, and community outreach programs can also play a vital role in educating the public about the importance of proper food handling and storage practices.

Strategies aimed at blocking bacterial establishment on surfaces often exploit stimuli to control the genes involved in biofilm formation.^143,144^ Understanding the molecular mechanisms involved in this process can help identify potential targets for the development of anti-biofilm agents. Molecular biology techniques, such as genome sequencing and transcriptomics, can be used to identify genes involved in biofilm formation and develop drugs that specifically target these genes or their products, leading to more effective therapy. On the contrary, mature biofilms can be dispersed by breaking off biofilm assemblies and promoting detachment. This requires disrupting EPS polymers by enzymes or programming cells to disperse.^145^

Given the challenge to conventional antimicrobial therapies due to increased resistance and genetic exchange of biofilm, a therapeutic approach that increases sensitivity to current methods or combines various modes of action with low cost is necessary.

## Summary

The biofilm lifestyle of *Salmonella* enables its persistence in the environment and in medical, veterinary, and industrial settings; it is a natural attribute, with the bacteria being significantly more widespread in its biofilm-producing form than in the planktonic form. The main reasons for this are enhanced tolerance to nutrient deficiencies and increased resistance to aggressive environmental conditions, including antimicrobial treatments. Indeed, infections caused by biofilm-forming bacteria require a substantially more intensive combination of antibiotics. Consequently, *Salmonella* becomes resistant to the medications being used, posing a threat to public health.

In this work, we have discussed the background of the factors regulating the main resistance mechanisms of *Salmonella* biofilms, with a particular emphasis on molecular agents. We have summarized the current knowledge of advancements in research dedicated to antibiotic-resistance-associated genes overexpressed in biofilms and described their role in *Salmonella* pathogenicity. Although much attention has been paid to this topic, there are still important scientific concerns that remain unanswered.

There are already known proteins directly associated with antibiotic resistance, including multiple resistance, transporters, xenobiotic degradation, and stress response proteins, while the roles of others, such as those involved in energy metabolism and outer membrane proteins or enzymes, are not primarily associated with enhanced treatment tolerance. Dozens of genes that encode these important proteins have been identified as upregulated in biofilms of *Enterobacteriaceae*. Although these genes or their homologs are present in *Salmonella*, most of the investigations presented transcriptomic data only and did not report their role in *Salmonella* pathogenicity. In addition, their biological functions were primarily validated in *E. coli* strains; therefore, we can only assume that their roles in *Salmonella* are analogous. We anticipate that this research will be broadened with comprehensive laboratory experiments consisting of the generation of deletion mutants and functional analysis using eukaryotic cell lines and various abiotic surfaces being utilized by *Salmonella*. There is no doubt that a large-scale xenobiotic resistance analysis should be performed to identify and study the antibiotic resistance profile of *Salmonella*. Another crucial factor that needs to be considered in future investigations is the serotype. It was previously shown that the outcomes can significantly differ due to different genetic backgrounds, such as single nucleotide polymorphisms (SNPs) in virulence factor-coding sequences. Thus, we believe that information obtained from a comparative analysis of representative strains would be beneficial.

Due to the increasing number of antibiotic-resistant biofilm-forming *Salmonella*, treatment of salmonellosis is impeded. This has led to the overuse of antibiotic-based therapies, especially in underdeveloped countries. As a result, selective pressure from antimicrobial therapy and antibiotic resistance crises have been observed worldwide. According to the developed models, antimicrobial resistance can cause 10 million deaths annually in the human population worldwide by 2050. Therefore, understanding the molecular patterns of biofilm formation, as well as defining the antibiotic resistance profile of biofilms under various environmental conditions, is required for the development of antibiofilm strategies. Considering the zoonotic potential of *Salmonella*, it is essential for both scientific research institutions and the food industry to develop techniques that effectively prevent biofilm formation and remove mature biofilms from the environment, food, and medical devices.

## Data Availability

Data sharing not applicable – no new data generated.

## References

[1] Havelaar AH, Kirk MD, Torgerson PR, Gibb HJ, Hald T, Lake RJ, Praet N, Bellinger DC, de Silva NR, Gargouri N, et al. World health organization global estimates and regional comparisons of the burden of foodborne disease in 2010. PLoS Med. 2015;12(12):e1001923. doi:10.1371/journal.pmed.1001923. 26633896 PMC4668832

[2] Ryan MP, O’Dwyer J, Adley CC. Evaluation of the complex nomenclature of the clinically and veterinary significant pathogen Salmonella. Biomed Res Int. 2017;2017 doi:10.1155/2017/3782182. PMC5429938 28540296

[3] Uzzau S, Brown DJ, Wallis T, Rubino S, Leori G, Bernard S, Casadesús J, Platt DJ, Olsen JE. Host adapted serotypes of Salmonella enterica. Epidemiol Infect. 2000;125(2):229–22. doi:10.1017/S0950268899004379. 11117946 PMC2869595

[4] Zhang S, Kingsley RA, Santos RL, Andrews-Polymenis H, Raffatellu M, Figueiredo J, Nunes J, Tsolis RM, Adams LG, Bäumler AJ, et al. Molecular pathogenesis of Salmonella enterica serotype typhimurium-induced diarrhea. Infect Immun. 2003;71(1):1–12. doi:10.1128/IAI.71.1.1-12.2003. 12496143 PMC143292

[5] Gal-Mor O. Persistent infection and long-term carriage of typhoidal and nontyphoidal salmonellae. Clin Microbiol Rev. 2019;32(1). doi:10.1128/CMR.00088-18. PMC6302356 30487167

[6] European Food Safety Authority and European Centre for Disease Prevention and Control (EFSA and ECDC). The European Union summary report on trends and sources of zoonoses, zoonotic agents and food-borne outbreaks in 2017. EFSA J. 2018;16(12). doi:10.2903/j.efsa.2018.5500. PMC7009540 32625785

[7] Vandenberg O, Nyarukweba DZ, Ndeba PM, Hendriksen RS, Barzilay EJ, Schirvel C, Bisimwa BB, Collard J-M, Aidara Kane A, Aarestrup FM, et al. Microbiologic and clinical features of salmonella species isolated from bacteremic children in eastern democratic republic of congo. Pediatr Infect Dis J. 2010;29(6):504–510. doi:10.1097/INF.0b013e3181cd615a. 20104200

[8] Costerton JW, Geesey GG, Cheng KJ. How bacteria stick. Sci Am. 1978;238(1):86–95. doi:10.1038/scientificamerican0178-86. 635520

[9] Harrell JE, Hahn MM, D’Souza SJ, Vasicek EM, Sandala JL, Gunn JS, McLachlan JB. Salmonella biofilm formation, chronic infection, and immunity within the intestine and hepatobiliary tract. Front Cell Infect Microbiol. 2021;10. doi:10.3389/fcimb.2020.624622. PMC7885405 33604308

[10] Landini P, Antoniani D, Burgess JG, Nijland R. Molecular mechanisms of compounds affecting bacterial biofilm formation and dispersal. Appl Microbiol Biotechnol. 2010;86(3):813–823. Preprint at. doi:10.1007/s00253-010-2468-8. 20165945

[11] Flemming HC, Wingender J. The biofilm matrix. Nat Rev Microbiol. 2010;8(9):623–633. doi:10.1038/nrmicro2415. 20676145

[12] Holden ER, Yasir M, Turner AK, Charles IG, Webber MA. Comparison of the genetic basis of biofilm formation between Salmonella Typhimurium and Escherichia coli. Microb Genom. 2022;8(11). doi:10.1099/mgen.0.000885. PMC9836088 36326671

[13] De la Fuente-Núñez C, Reffuveille F, Fernández L, Hancock REW. Bacterial biofilm development as a multicellular adaptation: antibiotic resistance and new therapeutic strategies. Curr Opin Microbiol. 2013;16(5):580–589. doi:10.1016/j.mib.2013.06.013. 23880136

[14] Mah TF. Biofilm-specific antibiotic resistance. Future Microbiol. 2012;7(9):1061–1072. doi:10.2217/fmb.12.76. 22953707

[15] Tursi SA, Tükel Ç. Curli-containing enteric biofilms inside and out: matrix composition, immune recognition, and disease implications. Microbiol Mol Biol R. 2018;82(4). doi:10.1128/MMBR.00028-18. PMC6298610 30305312

[16] Percival SL, Suleman L, Vuotto C, Donelli G. Healthcare-associated infections, medical devices and biofilms: risk, tolerance and control. J Med Microbiol. 2015;64(4):323–334. doi:10.1099/jmm.0.000032. 25670813

[17] Rabin N, Zheng Y, Opoku-Temeng C, Du Y, Bonsu E, Sintim HO. Biofilm formation mechanisms and targets for developing antibiofilm agents. Future Med Chem. 2015;7(4):493–512. doi:10.4155/fmc.15.6. 25875875

[18] MacKenzie KD, Palmer MB, Köster WL, White AP. Examining the link between biofilm formation and the ability of pathogenic Salmonella strains to colonize multiple host species. Front Vet Sci. 2017;4: doi:10.3389/fvets.2017.00138. PMC5581909 29159172

[19] Flemming HC, Wingender J, Szewzyk U, Steinberg P, Rice SA, Kjelleberg S. Biofilms: an emergent form of bacterial life. Nat Rev Microbiol. 2016;14(9):563–575. doi:10.1038/nrmicro.2016.94. 27510863

[20] Lowe JA. Chapter 13. Mechanisms of antibiotic resistance. 1982. p. 119–127. doi:10.1016/S0065-7743(08)60495-9.

[21] Poole K. Bacterial stress responses as determinants of antimicrobial resistance. J Antimicrob Chemother. 2012;67(9):2069–2089. doi:10.1093/jac/dks196. 22618862

[22] Stewart PS. Diffusion in biofilms. J Bacteriol. 2003;185(5):1485–1491. doi:10.1128/JB.185.5.1485-1491.2003. 12591863 PMC148055

[23] Behbahani SB, Kiridena SD, Wijayaratna UN, Taylor C, Anker JN, Tzeng TRJ. pH variation in medical implant biofilms: causes, measurements, and its implications for antibiotic resistance. Front Microbiol. 2022;13. doi:10.3389/fmicb.2022.1028560. PMC9659913 36386694

[24] Stewart PS. Mechanisms of antibiotic resistance in bacterial biofilms. Int J Med Microbiol. 2002;292(2):107–113. doi:10.1078/1438-4221-00196. 12195733

[25] Zogaj X, Nimtz M, Rohde M, Bokranz W, Römling U. The multicellular morphotypes of Salmonella typhimurium and Escherichia coli produce cellulose as the second component of the extracellular matrix. Mol Microbiol. 2001;39(6):1452–1463. doi:10.1046/j.1365-2958.2001.02337.x. 11260463

[26] Steenackers H, Hermans K, Vanderleyden J, De Keersmaecker SCJ. Salmonella biofilms: an overview on occurrence, structure, regulation and eradication. Food Res Int. 2012;45(2):502–531. doi:10.1016/j.foodres.2011.01.038.

[27] Adcox HE, Vasicek EM, Dwivedi V, Hoang KV, Turner J, Gunn JS. Salmonella extracellular matrix components influence biofilm formation and gallbladder colonization. Infect Immun. 2016;84(11):3243–3251. doi:10.1128/IAI.00532-16. 27600501 PMC5067756

[28] Zogaj X, Bokranz W, Nimtz M, Römling U. Production of cellulose and curli fimbriae by members of the family Enterobacteriaceae isolated from the human gastrointestinal tract. Infect Immun. 2003;71(7):4151–4158. doi:10.1128/IAI.71.7.4151-4158.2003. 12819107 PMC162016

[29] Hahn MM, González JF, Gunn JS. Salmonella biofilms tolerate hydrogen peroxide by a combination of extracellular polymeric substance barrier function and catalase enzymes. Front Cell Infect Microbiol. 2021;11. doi:10.3389/fcimb.2021.683081. PMC8171120 34095002

[30] Gibson DL, White AP, Snyder SD, Martin S, Heiss C, Azadi P, Surette M, Kay WW. Salmonella produces an O-antigen capsule regulated by AgfD and important for environmental persistence. J Bacteriol. 2006;188(22):7722–7730. doi:10.1128/JB.00809-06. 17079680 PMC1636306

[31] Rutherford ST, Bassler BL. Bacterial quorum sensing: its role in virulence and possibilities for its control. Cold Spring Harb Perspect Med. 2012;2(11):a012427–a012427. doi:10.1101/cshperspect.a012427. 23125205 PMC3543102

[32] Janssens JCA, Metzger K, Daniels R, Ptacek D, Verhoeven T, Habel LW, Vanderleyden J, De Vos DE, De Keersmaecker SCJ. Synthesis of N-acyl homoserine lactone analogues reveals strong activators of SdiA, the Salmonella enterica serovar typhimurium LuxR homologue. Appl Environ Microbiol. 2007;73(2):535–544. doi:10.1128/AEM.01451-06. 17085703 PMC1796990

[33] Choi J, Shin D, Ryu S. Implication of quorum sensing in Salmonella enterica serovar typhimurium virulence: the luxS gene is necessary for expression of genes in pathogenicity island 1. Infect Immun. 2007;75(10):4885–4890. doi:10.1128/IAI.01942-06. 17620352 PMC2044537

[34] Breijyeh Z, Jubeh B, Karaman R. Resistance of gram-negative bacteria to current antibacterial agents and approaches to resolve it. Molecules. 2020;25(6):1340. doi:10.3390/molecules25061340. 32187986 PMC7144564

[35] Ying YC, Bian HS, Tan TMC, Mattmann ME, Geske GD, Igarashi J, Hatano T, Suga H, Blackwell HE, Chua KL, et al. Control of quorum sensing by a Burkholderia pseudomallei multidrug efflux pump. J Bacteriol. 2007;189(11):4320–4324. doi:10.1128/JB.00003-07. 17384185 PMC1913402

[36] Buckley AM, Webber MA, Cooles S, Randall LP, La Ragione RM, Woodward MJ, Piddock LJV. The AcrAB-TolC efflux system of Salmonella enterica serovar Typhimurium plays a role in pathogenesis. Cell Microbiol. 2006;8(5):847–856. doi:10.1111/j.1462-5822.2005.00671.x. 16611233

[37] Nishino K, Nikaido E, Yamaguchi A. Regulation and physiological function of multidrug efflux pumps in Escherichia coli and Salmonella. Biochim Biophys Acta Proteins Proteomics. 2009;1794(5):834–843. doi:10.1016/j.bbapap.2009.02.002. 19230852

[38] Martins M, McCusker M, Amaral L, Fanning S. Mechanisms of antibiotic resistance in Salmonella: efflux pumps, genetics, quorum sensing and biofilm formation. Lett Drug Des Discov. 2011;8(2):114–123. doi:10.2174/157018011794183770.

[39] Fauvart M, de Groote VN, Michiels J. Role of persister cells in chronic infections: clinical relevance and perspectives on anti-persister therapies. J Med Microbiol. 2011;60(6):699–709. doi:10.1099/jmm.0.030932-0. 21459912

[40] Tezel BU, Akçelik N, Yüksel FN, Karatuğ NT, Akçelik M. Effects of sub-MIC antibiotic concentrations on biofilm production of Salmonella Infantis. Biotechnol Biotechnol Equip. 2016;30(6):1184–1191. doi:10.1080/13102818.2016.1224981.

[41] Narasanna R. Influence of subinhibitory-concentration (sub-MIC) Cefetoxime on biofilm formation. SEM study of ESBL-producing Salmonella typhi. J Microbiol Infect Dis. 2017;7(2):67–74. doi:10.5799/jmid.328786.

[42] Majtán J, Majtánová Ľ, Xu M, Majtán V. In vitro effect of subinhibitory concentrations of antibiotics on biofilm formation by clinical strains of Salmonella enterica serovar Typhimurium isolated in Slovakia. J Appl Microbiol. 2008;104(5):1294–1301. doi:10.1111/j.1365-2672.2007.03653.x. 18028358

[43] Koutsolioutsou A, Martins EA, White DG, Levy SB, Demple B. A soxRS-constitutive mutation contributing to antibiotic resistance in a clinical isolate of Salmonella enterica (Serovar typhimurium). Antimicrob Agents Chemother. 2001;45(1):38–43. doi:10.1128/AAC.45.1.38-43.2001. 11120941 PMC90236

[44] O’Regan E, Quinn T, Pagès J-M, McCusker M, Piddock L, Fanning S. Multiple regulatory pathways associated with high-level ciprofloxacin and multidrug resistance in Salmonella enterica serovar eNteritidis: involvement of ramA and other global regulators. Antimicrob Agents Chemother. 2009;53(3):1080–1087. doi:10.1128/AAC.01005-08. 19104017 PMC2650546

[45] Nonaka L, Maruyama F, Miyamoto M, Miyakoshi M, Kurokawa K, Masuda M. Novel conjugative transferable multiple drug resistance plasmid pAQU1 from *Photobacterium damselae* subsp. *damselae* isolated from marine aquaculture environment. Microb Environ. 2012;27(3):263–272. doi:10.1264/jsme2.ME11338. PMC4036041 22446310

[46] Sugimoto Y, Suzuki S, Nonaka L, Boonla C, Sukpanyatham N, Chou HY, Wu JH. The novel mef(C)–mph(G) macrolide resistance genes are conveyed in the environment on various vectors. J Glob Antimicrob Resist. 2017;10:47–53. doi:10.1016/j.jgar.2017.03.015. 28689921

[47] Yaron S, Kolling GL, Simon L, Matthews KR. Vesicle-mediated transfer of virulence genes from Escherichia coli O157: h7 to other enteric bacteria. Appl Environ Microbiol. 2000;66(10):4414–4420. doi:10.1128/AEM.66.10.4414-4420.2000. 11010892 PMC92318

[48] Bai X, Nakatsu CH, Bhunia AK. Bacterial biofilms and their implications in pathogenesis and food safety. Foods. 2021;10(9):2117. doi:10.3390/foods10092117. 34574227 PMC8472614

[49] Gunn JS, Marshall JM, Baker S, Dongol S, Charles RC, Ryan ET. Salmonella chronic carriage: epidemiology, diagnosis, and gallbladder persistence. Trends Microbiol. 2014;22(11):648–655. doi:10.1016/j.tim.2014.06.007. 25065707 PMC4252485

[50] Schembri MA, Kjærgaard K, Klemm P. Global gene expression in Escherichia coli biofilms. Mol Microbiol. 2003;48(1):253–267. doi:10.1046/j.1365-2958.2003.03432.x. 12657059

[51] Wang H, Zhang X, Dong Y, Xu X, Zhou G. Insights into the transcriptome profile of mature biofilm of Salmonella Typhimurium on stainless steels surface. Food Res Int. 2015;77:378–384. doi:10.1016/j.foodres.2015.08.034.

[52] Ghosh S, Lahiri D, Nag M, Sarkar T, Pati S, Edinur HA, Kumar M, Mohd Zain MRA, Ray RR. Precision targeting of food biofilm-forming genes by microbial scissors: cRISPR-Cas as an effective modulator. Front Microbiol. 2022;13: doi:10.3389/fmicb.2022.964848. PMC9396135 36016778

[53] Steenackers HP, Parijs I, Foster KR, Vanderleyden J, Banin E. Experimental evolution in biofilm populations. FEMS Microbiol Rev. 2016;40(3):373–397. doi:10.1093/femsre/fuw002. 26895713 PMC4852284

[54] Coenye T, Bové M, Bjarnsholt T. Biofilm antimicrobial susceptibility through an experimental evolutionary lens. NPJ Biofilms Microbiomes. 2022;8(1):82. doi:10.1038/s41522-022-00346-4. 36257971 PMC9579162

[55] Fuentes-Hernández A, Hernández-Koutoucheva A, Muñoz AF, Palestino RD, Peña-Miller R. Diffusion-driven enhancement of the antibiotic resistance selection window. J R Soc Interface. 2019;16(158):20190363. doi:10.1098/rsif.2019.0363. 31506045 PMC6769300

[56] Hegreness M, Shoresh N, Damian D, Hartl D, Kishony R. Accelerated evolution of resistance in multidrug environments. Proc Natl Acad Sci USA. 2008;105(37):13977–13981. doi:10.1073/pnas.0805965105. 18779569 PMC2544564

[57] Santos-Lopez A, Marshall CW, Scribner MR, Snyder DJ, Cooper VS. Evolutionary pathways to antibiotic resistance are dependent upon environmental structure and bacterial lifestyle. Elife. 2019;8. doi:10.7554/eLife.47612. PMC6814407 31516122

[58] Ahmed MN, Porse A, Sommer MOA, Høiby N, Ciofu O. Evolution of antibiotic resistance in biofilm and planktonic pseudomonas aeruginosa populations exposed to subinhibitory levels of ciprofloxacin. Antimicrob Agents Chemother. 2018;62(8). doi:10.1128/AAC.00320-18. PMC6105853 29760140

[59] Ahmed MN, Abdelsamad A, Wassermann T, Porse A, Becker J, Sommer MOA, Høiby N, Ciofu O. The evolutionary trajectories of P. aeruginosa in biofilm and planktonic growth modes exposed to ciprofloxacin: beyond selection of antibiotic resistance. NPJ Biofilms Microbiomes. 2020;6(1). doi:10.1038/s41522-020-00138-8. PMC7381665 32709907

[60] MacKenzie KD, Wang Y, Shivak DJ, Wong CS, Hoffman LJL, Lam S, Kröger C, Cameron ADS, Townsend HGG, Köster W, et al. Bistable expression of CsgD in Salmonella enterica serovar typhimurium connects virulence to persistence. Infect Immun. 2015;83(6):2312–2326. doi:10.1128/IAI.00137-15. 25824832 PMC4432751

[61] Lories B, Roberfroid S, Dieltjens L, De Coster D, Foster KR, Steenackers HP. Biofilm bacteria use stress responses to detect and respond to competitors. Curr Biol. 2020;30(7):1231–1244.e4. doi:10.1016/j.cub.2020.01.065. 32084407 PMC7322538

[62] Zou Y, Woo J, Ahn J. Cellular and molecular responses of Salmonella Typhimurium to antimicrobial-induced stresses during the planktonic-to-biofilm transition. Lett Appl Microbiol. 2012;55(4):274–282. doi:10.1111/j.1472-765X.2012.03288.x. 22803575

[63] Chin KCJ, Taylor TD, Hebrard M, Anbalagan K, Dashti MG, Phua KK. Transcriptomic study of Salmonella enterica subspecies enterica serovar Typhi biofilm. Bmc Genom. 2017;18(1). doi:10.1186/s12864-017-4212-6. PMC5664820 29089020

[64] Orme R, Douglas CWI, Rimmer S, Webb M. Proteomic analysis of Escherichia coli biofilms reveals the overexpression of the outer membrane protein OmpA. Proteomics. 2006;6(15):4269–4277. doi:10.1002/pmic.200600193. 16888722

[65] Hamilton S, Bongaerts RJ, Mulholland F, Cochrane B, Porter J, Lucchini S, Lappin-Scott HM, Hinton JC. The transcriptional programme of Salmonella enterica serovar Typhimurium reveals a key role for tryptophan metabolism in biofilms. Bmc Genom. 2009;10(1). doi:10.1186/1471-2164-10-599. PMC2805695 20003355

[66] Tabak M, Scher K, Hartog E, Romling U, Matthews KR, Chikindas ML, Yaron S. Effect of triclosan on Salmonella typhimurium at different growth stages and in biofilms. FEMS Microbiol Lett. 2007;267(2):200–206. doi:10.1111/j.1574-6968.2006.00547.x. 17156099

[67] Barbosa TM, Levy SB. Differential expression of over 60 chromosomal genes in Escherichia coli by constitutive expression of MarA. J Bacteriol. 2000;182(12):3467–3474. doi:10.1128/JB.182.12.3467-3474.2000. 10852879 PMC101932

[68] Pasqua M, Grossi M, Zennaro A, Fanelli G, Micheli G, Barras F, Colonna B, Prosseda G. The varied role of efflux pumps of the mfs family in the interplay of bacteria with animal and plant cells. Microorganisms. 2019;7(9):285. doi:10.3390/microorganisms7090285. 31443538 PMC6780985

[69] Yamasaki S, Nagasawa S, Fukushima A, Hayashi-Nishino M, Nishino K. Cooperation of the multidrug efflux pump and lipopolysaccharides in the intrinsic antibiotic resistance of Salmonella enterica serovar Typhimurium. J Antimicrob Chemother. 2013;68(5):1066–1070. doi:10.1093/jac/dks528. 23378414 PMC3625434

[70] Nishino K, Latifi T, Groisman EA. Virulence and drug resistance roles of multidrug efflux systems of Salmonella enterica serovar Typhimurium. Mol Microbiol. 2006;59(1):126–141. doi:10.1111/j.1365-2958.2005.04940.x. 16359323

[71] Baugh S, Ekanayaka AS, Piddock LJV, Webber MA. Loss of or inhibition of all multidrug resistance efflux pumps of Salmonella enterica serovar Typhimurium results in impaired ability to form a biofilm. J Antimicrob Chemother. 2012;67(10):2409–2417. doi:10.1093/jac/dks228. 22733653

[72] Andersen JL, He G-X, Kakarla P, Kc R, Kumar S, Lakra W, Mukherjee M, Ranaweera I, Shrestha U, Tran T, et al. Multidrug efflux pumps from Enterobacteriaceae, Vibrio cholerae and Staphylococcus aureus bacterial food pathogens. Int J Env Res Pub He. 2015;12(2):1487–1547. doi:10.3390/ijerph120201487. PMC4344678 25635914

[73] Kapach G, Nuri R, Schmidt C, Danin A, Ferrera S, Savidor A, Gerlach RG, Shai Y. Loss of the periplasmic chaperone skp and mutations in the efflux pump AcrAB-TolC play a role in acquired resistance to antimicrobial peptides in Salmonella typhimurium. Front Microbiol. 2020;11. doi:10.3389/fmicb.2020.00189. PMC7075815 32210923

[74] Hayashi M, Tabata K, Yagasaki M, Yonetani Y. Effect of multidrug-efflux transporter genes on dipeptide resistance and overproduction in Escherichia coli. FEMS Microbiol Lett. 2010;304(1):12–19. doi:10.1111/j.1574-6968.2009.01879.x. 20067529

[75] Hancock V, Klemm P. Global gene expression profiling of asymptomatic bacteriuria Escherichia coli during biofilm growth in human urine. Infect Immun. 2007;75(2):966–976. doi:10.1128/IAI.01748-06. 17145952 PMC1828481

[76] Nishino K, Yamaguchi A. Analysis of a complete library of putative drug transporter genes in Escherichia coli. J Bacteriol. 2001;183(20):5803–5812. doi:10.1128/JB.183.20.5803-5812.2001. 11566977 PMC99656

[77] Boyd D, Peters GA, Cloeckaert A, Boumedine KS, Chaslus-Dancla E, Imberechts H, Mulvey MR. Complete nucleotide sequence of a 43-kilobase genomic island associated with the multidrug resistance region of Salmonella enterica serovar typhimurium DT104 and its identification in phage type DT120 and serovar agona. J Bacteriol. 2001;183(19):5725–5732. doi:10.1128/JB.183.19.5725-5732.2001. 11544236 PMC95465

[78] Villagra NA, Fuentes JA, Jofre MR, Hidalgo AA, Garcia P, Mora GC. The carbon source influences the efflux pump-mediated antimicrobial resistance in clinically important Gram-negative bacteria. J Antimicrob Chemother. 2012;67(4):921–927. doi:10.1093/jac/dkr573. 22258924

[79] Marsh D, Henderson PJF. Specific spin labelling of the sugar-H+ symporter, GalP, in cell membranes of Escherichia coli: site mobility and overall rotational diffusion of the protein. Biochim Biophys Acta Biomembr. 2001;1510(1–2):464–473. doi:10.1016/S0005-2736(00)00377-1. 11342180

[80] Postma PW. Galactose transport in Salmonella typhimurium. J Bacteriol. 1977;129(2):630–639. doi:10.1128/jb.129.2.630-639.1977. 190207 PMC234992

[81] Weston N, Sharma P, Ricci V, Piddock LJV. Regulation of the AcrAB-TolC efflux pump in Enterobacteriaceae. Res Microbiol. 2018;169(7–8):425–431. doi:10.1016/j.resmic.2017.10.005. 29128373

[82] Du D, Wang Z, James NR, Voss JE, Klimont E, Ohene-Agyei T, Venter H, Chiu W, Luisi BF. Structure of the AcrAB–TolC multidrug efflux pump. Nature. 2014;509(7501):512–515. doi:10.1038/nature13205. 24747401 PMC4361902

[83] Horiyama T, Yamaguchi A, Nishino K. TolC dependency of multidrug efflux systems in Salmonella enterica serovar Typhimurium. J Antimicrob Chemother. 2010;65(7):1372–1376. doi:10.1093/jac/dkq160. 20495209

[84] Hwang J, Park S-H, Lee CW, Do H, Shin SC, Kim H-W, Lee SG, Park HH, Kwon S, Lee JH, et al. Crystal structure of a MarR family protein from the psychrophilic bacterium Paenisporosarcina sp. TG-14 in complex with a lipid-like molecule. IUCrJ. 2021;8(5):842–852. doi:10.1107/S2052252521005704. PMC8420766 34584745

[85] Martin RG, Rosner JL. Genomics of the marA/soxS/rob regulon of Escherichia coli: identification of directly activated promoters by application of molecular genetics and informatics to microarray data. Mol Microbiol. 2002;44(6):1611–1624. doi:10.1046/j.1365-2958.2002.02985.x. 12067348

[86] Beggs GA, Brennan RG, Arshad M. MarR family proteins are important regulators of clinically relevant antibiotic resistance. Protein Sci. 2020;29(3):647–653. doi:10.1002/pro.3769. 31682303 PMC7020996

[87] Perera IC, Grove A. Molecular mechanisms of ligand-mediated attenuation of DNA binding by MarR family transcriptional regulators. J Mol Cell Biol. 2010;2(5):243–254. doi:10.1093/jmcb/mjq021. 20716550

[88] Prieto AI, Herández SB, Cota I, Pucciarelli MG, Orlov Y, Ramos-Morales F, García-Del Portillo F, Casadesus J. Roles of the outer membrane protein asmA of Salmonella enterica in the control of marRAB expression and invasion of epithelial cells. J Bacteriol. 2009;191(11):3615–3622. doi:10.1128/JB.01592-08. 19346309 PMC2681915

[89] Feng Z, Liu D, Wang L, Wang Y, Zang Z, Liu Z, Song B, Gu L, Fan Z, Yang S, et al. A putative efflux transporter of the ABC family, YbhFSR, in Escherichia coli functions in tetracycline efflux and Na+(Li+)/H+ Transport. Front Microbiol. 2020;11. doi:10.3389/fmicb.2020.00556. PMC7190983 32390957

[90] Choi U, Lee CR. Distinct roles of outer membrane porins in antibiotic resistance and membrane integrity in Escherichia coli. Front Microbiol. 2019;10. doi:10.3389/fmicb.2019.00953. PMC6503746 31114568

[91] Pagès JM, James CE, Winterhalter M. The porin and the permeating antibiotic: a selective diffusion barrier in Gram-negative bacteria. Nat Rev Microbiol. 2008;6(12):893–903. doi:10.1038/nrmicro1994. 18997824

[92] Moya-Torres A, Mulvey MR, Kumar A, Oresnik IJ, Brassinga AKC. The lack of OmpF, but not OmpC, contributes to increased antibiotic resistance in Serratia marcescens. Microbiology (UK). 2014;160(9):1882–1892. doi:10.1099/mic.0.081166-0. 25015362

[93] Villarreal JM, Becerra-Lobato N, Rebollar-Flores JE, Medina-Aparicio L, Carbajal-Gómez E, Zavala-García ML, Vázquez A, Gutiérrez-Ríos RM, Olvera L, Encarnación S, et al. The Salmonella enterica serovar Typhi ltrR-ompR-ompC-ompF genes are involved in resistance to the bile salt sodium deoxycholate and in bacterial transformation. Mol Microbiol. 2014;92(5):1005–1024. doi:10.1111/mmi.12610. 24720747

[94] Shi C, Li M, Muhammad I, Ma X, Chang Y, Li R, Li C, He J, Liu F. Combination of berberine and ciprofloxacin reduces multi-resistant Salmonella strain biofilm formation by depressing mRNA expressions of luxS, rpoE, and ompR. J Vet Sci. 2018;19(6):808. doi:10.4142/jvs.2018.19.6.808. 30304890 PMC6265579

[95] Perni S, Preedy EC, Landini P, Prokopovich P. Influence of csgD and ompR on nanomechanics, adhesion forces, and curli properties of E. coli. Langmuir. 2016;32(31):7965–7974. doi:10.1021/acs.langmuir.6b02342. 27434665

[96] Ziervogel BK, Roux B. The binding of antibiotics in OmpF porin. Structure. 2013;21(1):76–87. doi:10.1016/j.str.2012.10.014. 23201272 PMC3545085

[97] Matera G, Altuvia Y, Gerovac M, El Mouali Y, Margalit H, Vogel J. Global RNA interactome of Salmonella discovers a 5′ UTR sponge for the MicF small RNA that connects membrane permeability to transport capacity. Mol Cell. 2022;82(3):629–644.e4. doi:10.1016/j.molcel.2021.12.030. 35063132

[98] Medeiros AA, O’Brien TF, Rosenberg EY, Nikaido H. Loss of OmpC porin in a strain of salmonella typhimirium causes increased resistance to cephalosporins during therapy. J Infect Dis. 1987;156(5):751–757. doi:10.1093/infdis/156.5.751. 2821125

[99] Chowdhury AR, Mukherjee D, Singh AK, Chakravortty D. Loss of outer membrane protein a (OmpA) impairs the survival of Salmonella Typhimurium by inducing membrane damage in the presence of ceftazidime and meropenem. J Antimicrob Chemother. 2022;77(12):3376–3389. doi:10.1093/jac/dkac327. 36177811

[100] Smani Y, Fàbrega A, Roca I, Sánchez-Encinales V, Vila J, Pachón J. Role of OmpA in the multidrug resistance phenotype of Acinetobacter baumannii. Antimicrob Agents Chemother. 2014;58(3):1806–1808. doi:10.1128/AAC.02101-13. 24379205 PMC3957889

[101] Dupont M, James CE, Chevalier J, Pagès JM. An early response to environmental stress involves regulation of OmpX and OmpF, two enterobacterial outer membrane pore-forming proteins. Antimicrob Agents Chemother. 2007;51(9):3190–3198. doi:10.1128/AAC.01481-06. 17606680 PMC2043185

[102] Briones AC, Lorca D, Cofre A, Cabezas CE, Krüger GI, Pardo-Esté C, Baquedano MS, Salinas CR, Espinoza M, Castro-Severyn J, et al. Genetic regulation of the ompX porin of Salmonella Typhimurium in response to hydrogen peroxide stress. Biol Res. 2022;55(1). doi:10.1186/s40659-022-00377-3. PMC8862304 35193678

[103] Hirakawa H, Suzue K, Takita A, Kamitani W, Tomita H, Brodsky IE. Roles of ompx, an outer membrane protein, on virulence and flagellar expression in uropathogenic Escherichia coli. Infect Immun. 2021;89(6). doi:10.1128/IAI.00721-20. PMC8316124 33753414

[104] Wang Y, Yi L, Zhang J, Sun L, Wen W, Zhang C, Wang S. Functional analysis of superoxide dismutase of Salmonella typhimurium in serum resistance and biofilm formation. J Appl Microbiol. 2018;125(5):1526–1533. doi:10.1111/jam.14044. 29989280

[105] Stevanin TM, Poole RK, Demoncheaux EAG, Read RC. Flavohemoglobin Hmp protects Salmonella enterica serovar typhimurium from nitric oxide-related killing by human macrophages. Infect Immun. 2002;70(8):4399–4405. doi:10.1128/IAI.70.8.4399-4405.2002. 12117950 PMC128135

[106] Fukuda M, Takeda H, Kato HE, Doki S, Ito K, Maturana AD, Ishitani R, Nureki O. Structural basis for dynamic mechanism of nitrate/nitrite antiport by NarK. Nat Commun. 2015;6(1). doi:10.1038/ncomms8097. PMC4432589 25959928

[107] Jia W, Tovell N, Clegg S, Trimmer M, Cole J. A single channel for nitrate uptake, nitrite export and nitrite uptake by Escherichia coli NarU and a role for NirC in nitrite export and uptake. Biochem J. 2009;417(1):297–307. doi:10.1042/BJ20080746. 18691156

[108] Wand ME, Bock LJ, Bonney LC, Sutton JM. Mechanisms of increased resistance to chlorhexidine and cross-resistance to colistin following exposure of Klebsiella pneumoniae clinical isolates to chlorhexidine. Antimicrob Agents Chemother. 2017;61(1). doi:10.1128/AAC.01162-16. PMC5192135 27799211

[109] Sakai A, Nakanishi M, Yoshiyama K, Maki H. Impact of reactive oxygen species on spontaneous mutagenesis in Escherichia coli. Genes to Cells. 2006;11(7):767–778. doi:10.1111/j.1365-2443.2006.00982.x. 16824196

[110] Cosgrove K, Coutts G, Jonsson I-M, Tarkowski A, Kokai-Kun JF, Mond JJ, Foster SJ. Catalase (KatA) and alkyl hydroperoxide reductase (AhpC) have compensatory roles in peroxide stress resistance and are required for survival, persistence, and nasal colonization in Staphylococcus aureus. J Bacteriol. 2007;189(3):1025–1035. doi:10.1128/JB.01524-06. 17114262 PMC1797328

[111] Jacobson FS, Morgan RW, Christman MF, Ames BN. An alkyl hydroperoxide reductase from Salmonella typhimurium involved in the defense of DNA against oxidative damage. J Biol Chem. 1989;264(3):1488–1496. doi:10.1016/S0021-9258(18)94214-6. 2643600

[112] Parsonage D, Karplus PA, Poole LB. Substrate specificity and redox potential of AhpC, a bacterial peroxiredoxin. Proc Natl Acad Sci USA. 2008;105(24):8209–8214. doi:10.1073/pnas.0708308105. 18165315 PMC2448816

[113] Bollinger N, Hassett DJ, Iglewski BH, Costerton JW, McDermott TR. Gene expression in Pseudomonas aeruginosa: evidence of iron override effects on quorum sensing and biofilm-specific gene regulation. J Bacteriol. 2001;183(6):1990–1996. doi:10.1128/JB.183.6.1990-1996.2001. 11222597 PMC95094

[114] Jang IA, Kim J, Park W. Endogenous hydrogen peroxide increases biofilm formation by inducing exopolysaccharide production in Acinetobacter oleivorans DR1. Sci Rep. 2016;6(1). doi:10.1038/srep21121. PMC4756669 26884212

[115] Loewen PC, De Silva PM, Donald LJ, Switala J, Villanueva J, Fita I, Kumar A. KatG-Mediated oxidation leading to reduced susceptibility of bacteria to kanamycin. ACS Omega. 2018;3(4):4213–4219. doi:10.1021/acsomega.8b00356. 29732452 PMC5928485

[116] Zhu Y-Q, Zhu D-Y, Lu H-X, Yang N, Li G-P, Wang D-C. Purification and preliminary crystallographic studies of CutC, a novel copper homeostasis protein from shigella flexneri. Protein Pept Lett. 2005;12(8):823–826. doi:10.2174/0929866054864184. 16305556

[117] Grass G, Rensing C, Solioz M. Metallic copper as an antimicrobial surface. Appl Environ Microb. 2011;77(5):1541–1547. doi:10.1128/AEM.02766-10. PMC3067274 21193661

[118] Gupta SD, Lee BTO, Camakaris J, Wu HC. Identification of cutC and cutF (nlpE) genes involved in copper tolerance in Escherichia coli. J Bacteriol. 1995;177(15):4207–4215. doi:10.1128/jb.177.15.4207-4215.1995. 7635807 PMC177164

[119] Geslin C, Llanos J, Prieur D, Jeanthon C. The manganese and iron superoxide dismutases protect Escherichia coli from heavy metal toxicity. Res Microbiol. 2001;152(10):901–905. doi:10.1016/S0923-2508(01)01273-6. 11766965

[120] Calhoun LN, Kwon YM. The ferritin-like protein Dps protects Salmonella enterica serotype Enteritidis from the Fenton-mediated killing mechanism of bactericidal antibiotics. Int J Antimicrob Agents. 2011;37(3):261–265. doi:10.1016/j.ijantimicag.2010.11.034. 21295952

[121] Nair S, Finkel SE. Dps protects cells against multiple stresses during stationary phase. J Bacteriol. 2004;186(13):4192–4198. doi:10.1128/JB.186.13.4192-4198.2004. 15205421 PMC421617

[122] Almiron M, Link AJ, Furlong D, Kolter R. A novel DNA-binding protein with regulatory and protective roles in starved Escherichia coli. Genes Dev. 1992;6(12b):2646–2654. doi:10.1101/gad.6.12b.2646. 1340475

[123] Goto R, Miki T, Nakamura N, Fujimoto M, Okada N, Cloeckaert A. Salmonella Typhimurium PagP- and UgtL-dependent resistance to antimicrobial peptides contributes to the gut colonization. PLoS One. 2017;12(12):e0190095. doi:10.1371/journal.pone.0190095. 29267354 PMC5739500

[124] Shi Y, Cromie MJ, Hsu FF, Turk J, Groisman EA. PhoP-regulated Salmonella resistance to the antimicrobial peptides magainin 2 and polymyxin B. Mol Microbiol. 2004;53(1):229–241. doi:10.1111/j.1365-2958.2004.04107.x. 15225317

[125] Pardo-Esté C, Castro-Severyn J, Krüger GI, Cabezas CE, Briones AC, Aguirre C, Morales N, Baquedano MS, Sulbaran YN, Hidalgo AA, et al. The transcription factor ArcA Modulates Salmonella’s metabolism in response to neutrophil hypochlorous acid-mediated stress. Front Microbiol. 2019;10. doi:10.3389/fmicb.2019.02754. PMC6906141 31866961

[126] Zhang XS, García-Contreras R, Wood TK. YcfR (BhsA) influences Escherichia coli biofilm formation through stress response and surface hydrophobicity. J Bacteriol. 2007;189(8):3051–3062. doi:10.1128/JB.01832-06. 17293424 PMC1855844

[127] Salazar JK, Deng K, Tortorello ML, Brandl MT, Wang H, Zhang W. Genes ycfR, sirA and yigG contribute to the surface attachment of Salmonella enterica typhimurium and saintpaul to fresh produce. PLoS One. 2013;8(2):e57272. doi:10.1371/journal.pone.0057272. 23451197 PMC3579871

[128] Nobre LS, Al-Shahrour F, Dopazo J, Saraiva LM. Exploring the antimicrobial action of a carbon monoxide-releasing compound through whole-genome transcription profiling of Escherichia coli. Microbiology (NY). 2009;155:813–824. 10.1099/mic.0.023911-0 19246752

[129] Conter A, Gangneux C, Suzanne M, Gutierrez C. Survival of Escherichia coli during long-term starvation: effects of aeration, NaCl, and the rpoS and osmC gene products. Res Microbiol. 2001;152(1):17–26. doi:10.1016/S0923-2508(00)01164-5. 11281321

[130] Mongkolsuk S, Praituan W, Loprasert S, Fuangthong M, Chamnongpol S. Identification and characterization of a new organic hydroperoxide resistance (ohr) gene with a novel pattern of oxidative stress regulation from Xanthomonas campestris pv. phaseoli. J Bacteriol. 1998;180(10):2636–2643. doi:10.1128/JB.180.10.2636-2643.1998. 9573147 PMC107214

[131] Vickery K. Special Issue: microbial biofilms in healthcare: formation, prevention and treatment. Materials. 2019;12(12):2001. doi:10.3390/ma12122001. 31234513 PMC6630215

[132] Schulze A, Mitterer F, Pombo JP, Schild S. Biofilms by bacterial human pathogens: clinical relevance - Development, composition and regulation - Therapeutical strategies. Microb Cell. 2021;8(2):28–56. doi:10.15698/mic2021.02.741. 33553418 PMC7841849

[133] Madsen JS, Burmølle M, Hansen LH, Sørensen SJ. The interconnection between biofilm formation and horizontal gene transfer. FEMS Immunol Med Microbiol. 2012;65(2):183–195. doi:10.1111/j.1574-695X.2012.00960.x. 22444301

[134] Gamazo C, Solano C, Lasa I. Biofilm formation by Salmonella in food processing environments. In: Biofilms in the food and beverage industries. Elsevier; 2009. pp. 226–249. doi:10.1533/9781845697167.2.226

[135] Joseph B, Otta SK, Karunasagar I, Karunasagar I. Biofilm formation by Salmonella spp. On food contact surfaces and their sensitivity to sanitizers. Int J Food Microbiol. 2001;64(3):367–372. doi:10.1016/S0168-1605(00)00466-9. 11294359

[136] Liu H, Whitehouse CA, Li B. Presence and persistence of Salmonella in water: the impact on microbial quality of water and food safety. Front Public Health. 2018;6: doi:10.3389/fpubh.2018.00159. PMC5989457 29900166

[137] Liu Z, Adyel TM, Miao L, You G, Liu S, Hou J. Biofilm influenced metal accumulation onto plastic debris in different freshwaters. Environ Pollut. 2021;285:117646. doi:10.1016/j.envpol.2021.117646. 34380227

[138] Cloete TE, Jacobs L, Brözel VS. The chemical control of biofouling in industrial water systems. Biodegradation. 1998;9(1):23–37. doi:10.1023/A:1008216209206. 9807802

[139] Colclough A, Corander J, Sheppard SK, Bayliss SC, Vos M. Patterns of cross-resistance and collateral sensitivity between clinical antibiotics and natural antimicrobials. Evol Appl. 2019;12(5):878–887. doi:10.1111/eva.12762. 31080502 PMC6503891

[140] Roy R, Tiwari M, Donelli G, Tiwari V. Strategies for combating bacterial biofilms: a focus on anti-biofilm agents and their mechanisms of action. Virulence. 2018;9(1):522–554. doi:10.1080/21505594.2017.1313372. 28362216 PMC5955472

[141] Pogodin S, Hasan J, Baulin V, Webb H, Truong V, Phong Nguyen T, Boshkovikj V, Fluke C, Watson G, Watson J, et al. Biophysical model of bacterial cell interactions with nanopatterned cicada wing surfaces. Biophys J. 2013;104(4):835–840. doi:10.1016/j.bpj.2012.12.046. 23442962 PMC3576530

[142] Banat IM, De Rienzo MAD, Quinn GA. Microbial biofilms: biosurfactants as antibiofilm agents. Appl Microbiol Biotechnol. 2014;98(24):9915–9929. doi:10.1007/s00253-014-6169-6. 25359476

[143] Brackman G, Coenye T. Quorum sensing inhibitors as anti-biofilm agents. Curr Pharm Des. 2014;21(1):5–11. doi:10.2174/1381612820666140905114627. 25189863

[144] Ren D, Sims JJ, Wood TK. Inhibition of biofilm formation and swarming of Escherichia coli by (5Z)-4-bromo-5-(bromomethylene)-3- butyl-2(5H)-furanone. Environ Microbiol. 2001;3(11):731–736. doi:10.1046/j.1462-2920.2001.00249.x. 11846763

[145] Li X-H, Lee J-H. Antibiofilm agents: a new perspective for antimicrobial strategy. J Microbiol. 2017;55(10):753–766. doi:10.1007/s12275-017-7274-x. 28956348

